# Preclinical Evaluation of Safety, Pharmacokinetics, Efficacy, and Mechanism of Radioprotective Agent HL-003

**DOI:** 10.1155/2021/6683836

**Published:** 2021-02-19

**Authors:** Yahong Liu, Longfei Miao, Yuying Guo, Hongqi Tian

**Affiliations:** Tianjin Key Laboratory of Radiation Medicine and Molecular Nuclear Medicine, Institute of Radiation Medicine, Peking Union Medical College and Chinese Academy of Medical Science, Tianjin 300192, China

## Abstract

Amifostine is a radioprotector with high efficacy but poor safety, short half-life, no oral formulation, and poor compliance, which limits its application. With the increasing risk of exposure to radiation, the development of new radioprotective agents is critical. We previously synthesized a new amifostine derivative, the small molecule compound HL-003. In this study, we focused on evaluating the radioprotective properties of HL-003. Using the *in vitro* 2,2-diphenyl-1-picrylhydrazyl assay, we initially confirmed HL-003 as a strong antioxidant and demonstrated that its free radical scavenging activity was stronger than that of amifostine. Then, we performed an acute toxicity test, a 28-day toxicity test, a 30-day survival rate test, and a pharmacokinetic study, all of which provided aggregate evidence that HL-003 functioned as a small molecule radioprotector with high efficacy, a favorable safety profile, a long half-life, and oral administration. The intestinal radioprotective mechanism of HL-003 was explored in male C57 mice after abdominal irradiation by analyzing intestinal tissue samples with hematoxylin-eosin staining, immunohistochemistry, TUNEL staining, and immunofluorescence detection. The results showed that HL-003 protected intestinal DNA from radiation damage and suppressed the expression of phosphorylated histone H2AX, phosphorylated p53, and the apoptosis-related proteins caspase-8 and caspase-9, which contributed to maintaining the normal morphology of the small intestine and provided insights into the mechanism of radioprotection. Thus, HL-003 is a small molecule radioprotector with a potential application in radiation medicine.

## 1. Introduction

The civilian population is at an increased risk of radiation exposure due to the increased possibility of nuclear weapon explosion, destruction of nuclear facilities, exposure to radioactive materials, terrorist attacks, and nuclear accidents, all of which can have a catastrophic impact on public health [1]. Acute radiation syndrome (ARS) occurs when the intensity of the whole-body or partial-body irradiation is greater than 1 Gy. The main clinical manifestations are hematopoiesis (2–6 Gy), along with the gastrointestinal (6–8 Gy) and cerebrovascular syndrome (>8 Gy) [2–4]. In the past 60 years, despite significant scientific and technological advances in the development of safe, nontoxic, and effective radiation strategies for ARS, the U.S. Food and Drug Administration has not approved any drugs [5]. At present, radiation countermeasures can be divided into three categories: radioprotective agents administered before exposure to prevent damage, radiation mitigators applied shortly after radiation exposure but before the appearance of radiation symptoms to accelerate recovery or repair, and radiation therapeutics or treatments given after the onset of symptoms to stimulate repair or regeneration [6]. Our research was aimed at radioprotective agents.

Previous reports indicated that the mechanism of radiation injury is closely associated with the production of reactive oxygen species (ROS), causing oxidative damage to proteins, lipids, and DNA, and activates activating signal transduction pathways [7–9]. Thus, using antioxidants for radioprotection is a critical medical countermeasure. Amifostine is the most active small molecule radioprotector that was developed by Walter Reed Army Institute of Research in 1959 to protect soldiers against the damage caused by nuclear radiation [5, 10, 11]. However, its application is severely limited due to its short half-life, its administration by injection, the lack of an oral formulation, poor compliance, and serious side effects, including nausea, vomiting, and hypotension [12, 13].

To obtain radioprotectors with fewer side effects than amifostine, a variety of amino mercaptan analogs were designed and synthesized, including *β*-aminoethyl isothiourea (AET), N-acetyl-L-cysteine (NAC), N-2-mercaptopropionylglycine (MPG), diethyldithiocarbamate (DDC), 2,2-dimethylthiazolidine, and PrC-210 [14–16]. However, the radioprotective effect of these compounds still needs to be improved, except for PrC-210 [17]. Furthermore, although well-known natural antioxidants, such as curcumin, resveratrol, vitamin C, and red ginseng, have low toxicity and high tolerability, their radioprotection is less effective than that of amifostine [18–21]. Hence, there is an urgent need to develop oral small molecule radioprotectors with high efficiency and low toxicity.

We previously developed a new small molecule, HL-003, with 100% intellectual property rights [22]. In the current study, we initially used a cell-free system to investigate the antioxidant activity of HL-003 and its protective effect against oxidative damage of DNA. We then systematically evaluated the safety, efficacy, and pharmacokinetic (PK) characteristics of HL-003, including its ability to cross the blood-brain barrier (BBB). Finally, we studied the radioprotection mechanism of HL-003 in radiation-induced small intestinal injury. Our analysis indicated that the small molecule antioxidant HL-003 has a potential clinical value as a radioprotectant against ARS.

## 2. Materials and Methods

### 2.1. Animals

Male C57BL/6 mice (21–22 g), male ICR mice (21–22 g), and male SD rats (200–300 g) were purchased from SPF HFK Bioscience Co., Ltd. (Beijing, China), JOINN Laboratories (Beijing, China), and Charles River Laboratories (Beijing, China), respectively. All experimental animals were maintained in an accredited animal facility according to the guidelines of the National Animal Welfare Law of China and the NIH Guide for the Care and Use of Laboratory Animals. All animal procedures were approved by the Institutional Animal Care and Use Committee of the Institute of Radiation Medicine, Chinese Academy of Medical Science (permit number: 2017053).

### 2.2. 2,2-Diphenyl-1-picrylhydrazyl (DPPH) Free Radical Scavenging Test

DPPH (CAS No. 1898-664) was precisely weighed (6.4 mg) and fully dissolved in 32 mL anhydrous ethanol to prepare a stock solution for storage at 4°C. Before the experiment, the stock solution was diluted to 50 *μ*g/mL. HL-003 (10 mg) was dissolved with 1 mL ddH_2_O and twofold serially diluted to obtain working solutions of 10, 5, 2.5, 1.25, 0.625, 0.3125, 0.15625, 0.078125, and 0.0390625 mg/mL. Aliquots of 180 *μ*L DPPH ethanol solution were dispensed on a 96-well plate (No. 14319035; Corning Inc., Corning, NY, USA) and mixed with 20 *μ*L aliquots of HL-003 at different concentrations. Control samples contained 180 *μ*L ethanol solution mixed with 20 *μ*L HL-003 solution at different concentrations. Each sample was prepared and processed in triplicate. The test was performed by incubating the samples in the dark for 30 min at room temperature. The samples were analyzed by measuring the absorbance at 515 nm using a microplate reader (SM600, Shanghai Utrao Medical Instrument Co., Ltd.).

### 2.3. Acute Toxicity Test

A total of 88 male C57BL/6 mice were randomly assigned to the acute toxicity test groups. Sixty mice were divided into six HL-003 groups (10 mice per group). Each group received a different HL-003 dose (1200, 1600, 1800, 2000, 2400, and 3200 mg/kg) by oral gavage. Eighteen mice were treated with amifostine (No. 112901-68-5; Jiangsu Aikon Biopharmaceutical R&D Co., Ltd.). The mice were divided into three groups (6 mice per group), each of which received a different amifostine dose (600, 700, and 800 mg/kg) by oral gavage. In the control group (10 mice), oral gavage was performed to administer the HL-003 vehicle containing 85% of a 20% hydroxypropyl-*β*-cyclodextrin solution (HP-*β*-CD, No. 20190414; Zhiyuan Biotechnology) and 15% Solutol HS-15 (HF08982; Shenzhen hiboled Century Biotechnology Co., Ltd.). The toxicity of the treatments and the death of mice were monitored and recorded for 14 days postadministration. All surviving mice were euthanized at the end of the experiment.

### 2.4. Long-Term Toxicity Test

A total of 50 male C57BL/6 mice were randomly assigned to the long-term toxicity test groups. Forty mice were divided into four HL-003 groups (10 mice per group). For 28 days, each treatment group received a different HL-003 dose (800, 1000, 1200, and 1600 mg/kg) by oral gavage. The control group (10 mice) received the HL-003 vehicle solution for 28 days by oral gavage. The toxicity of the treatments and the death of mice were observed and recorded for 28 days. All surviving mice were euthanized at the end of the experiment.

### 2.5. Radioprotective Efficacy of Orally Administered HL-003 and Amifostine

Fifty-two male C57BL/6 mice were randomly divided into seven groups: the control group (*n* = 7), IR 8 Gy group (*n* = 7), HL-003 800 mg/kg (*n* = 7), HL-003 1200 mg/kg (*n* = 7), HL-003 1600 mg/kg (*n* = 7), amifostine 200 mg/kg (*n* = 7), and amifostine 500 mg/kg (*n* = 10). HL-003 was orally administered 1 h before the 8 Gy whole-body irradiation (WBI) in all HL-003 groups. In the amifostine groups, a dose of 200 or 500 mg/kg amifostine was orally administered 0.5 or 1 h before the 8 Gy WBI, respectively. In the IR 8 Gy group, the HL-003 vehicle solution was orally administered 1 h before the 8 Gy WBI. In the control group, the HL-003 vehicle solution was orally administered without radiation exposure. A ^137^Cs source enclosed in an Exposure Instrument Gammacell-40 (Atomic Energy of Canada Limited, Chalk River, ON, Canada) was used as the radiation source. The mice were continuously monitored for 30 days after irradiation. The death of mice was recorded, and the survival curve was generated using GraphPad Prism 6.0 software.

### 2.6. Screening for the Optimal Time to Perform Oral HL-003 Administration

Sixty male C57BL/6 mice were randomly divided into six groups (10 mice per group): the control group, IR 8 Gy group, HL-003 0.5 h group, HL-003 1 h group, HL-003 2 h group, and HL-003 4 h group. HL-003 (1600 mg/kg) was orally administered 0.5, 1, 2, or 4 h before the 8 Gy WBI. In the IR 8 Gy group, the HL-003 vehicle solution was orally administered 1 h before the 8 Gy WBI. In the control group, the HL-003 vehicle solution was orally administered without irradiation. The mice were continuously monitored for 30 days after irradiation. The death of mice was recorded, and the survival curve was generated using GraphPad Prism 6.0 software.

### 2.7. Radioprotective Efficacy of HL-003 at 10 Gy

Fifty male C57BL/6 mice were randomly divided into five groups (10 mice per group): the control group, IR 10 Gy group, HL-003 3 h group, HL-003 4 h group, and HL-003 6 h group. HL-003 (1600 mg/kg) was orally administered 3, 4, and 6 h before the 10 Gy WBI. In the IR 10 Gy group, the HL-003 vehicle solution was orally administered 4 h before the 10 Gy WBI. In the control group, the HL-003 vehicle solution was orally administered without irradiation. The mice were continuously monitored for 30 days after irradiation. The death of mice was recorded, and the survival curve was generated using GraphPad Prism 6.0 software.

### 2.8. PK Study of HL-003

Six male SD rats were randomly divided into two groups (3 rats per group), receiving a dose of either 100 mg/kg HL-003 by intravenous (i.v.) injection or 400 mg/kg HL-003 per os (p.o.). Blood samples were collected at 0.08, 0.25, 0.5, 1, 2, 4, 8, and 24 h after HL-003 administration (i.v. or p.o.). Aliquots of 50 *μ*L plasma were derived from each blood sample. The plasma aliquots were loaded on a 96-well plate and mixed with 250 *μ*L acetonitrile (WXBC4001V; Sigma-Aldrich, St. Louis, MO, USA). The plate was swirled and centrifuged at 400 rpm for 20 min. Each supernatant (150 *μ*L) was mixed with 150 *μ*L of 0.1% formic acid (C1728048; Aladdin) and swirled for 10 min. Aliquots of 10.0 *μ*L per sample were injected and analyzed by liquid chromatography-tandem mass spectrometry (LC/MS/MS, TQU335, Thermo Scientific, Wilmington, MA, USA).

### 2.9. BBB Permeability Evaluation of HL-003

At 0.25, 0.5, 1, 2, 3, 4, 6, and 8 h after male ICR mice received an oral dose of 400 mg/kg HL-003, whole blood aliquots of approximately 200 *μ*L were collected in blood collection vessels containing EDTA, and brain tissue samples were recovered by necropsy. The collected blood was placed on ice and centrifuged at 4000 rpm for 20 min. The plasma was separated into 1.5 mL EP tubes. After the mice were euthanized, the brains were quickly removed. The residual blood on the brain tissue was cleaned with ice water, and the residual water on the brain tissue was dried with dry medical gauze. After weighing, the brain and plasma samples were stored at -80°C for testing. Samples were analyzed by LC/MS/MS (TQU335, Thermo Scientific).

### 2.10. Analysis of the Protective Mechanism of HL-003 against Intestinal Irradiation

Nine male SD rats were randomly divided into three groups (3 rats per group): the control group, IR group, and HL-003+IR group. The rats in the IR group received 15 Gy abdominal irradiation (ABI). In the HL-003+IR group, a dose of 1600 mg/kg HL-003 was orally administered 4 h before the 15 Gy ABI. Five days after the IR, all rats were sacrificed to collect small intestinal tissue for mechanism analysis.

### 2.11. Hematoxylin and Eosin (He) Staining

Tissue samples of the small intestine were fixed in 4% paraformaldehyde, embedded in paraffin, and cut into 4 *μ*m sections. The sections were dewaxed with xylene, stained with HE, and analyzed with a microscope (Olympus America, Melville, NY, USA).

### 2.12. Immunohistochemistry Analysis

Tissue samples of the small intestine were fixed in 4% paraformaldehyde, embedded in paraffin, cut into 4 *μ*m sections, and dewaxed with xylene. The sections were evenly covered with goat serum and sealed for 30 min at room temperature. After discarding the sealing fluid, the slices were incubated overnight at 4°C with a primary antibody at an appropriate dilution, including anti-Lgr5 antibody (1 : 300, bs-20747R; Bioss), anti-lysozyme antibody (1 : 100, ab108508; Abcam), and anti-Ki67 antibody (1 : 300, ab15580; Abcam), followed by incubation with secondary antibodies conjugated to peroxidase for 50 min at room temperature. Subsequently, the sections were stained with 3,3′-diaminobenzidine (DAB) and hematoxylin for microscopic analysis (OLYMPUS DP26). Hematoxylin stained the nuclei blue, and DAB produced a brown stain for positive expression of the targeted proteins.

### 2.13. TUNEL Assay

Tissue samples of the small intestine were fixed in 4% paraformaldehyde, embedded in paraffin, cut into 4 *μ*m sections, and dewaxed with xylene. The sections were processed with a TUNEL kit (*In Situ* Cell Death Detection Kit, 11684817910; Roche), and the images were analyzed with a fluorescence microscope (OLYMPUS BX51).

### 2.14. Immunofluorescence Analysis

Tissue samples of the small intestine were fixed in 4% paraformaldehyde, embedded in paraffin, cut into 4 *μ*m sections, and dewaxed with xylene. The sections were evenly covered with goat serum and sealed for 30 min at room temperature. After discarding the sealing fluid, the slices were incubated overnight at 4°C with a primary antibody at an appropriate dilution, including anti-*γ*-H2AX antibody (1 : 100, bs-3185R; Bioss), anti-p53 antibody (1 : 100, ab26; Abcam), anti-caspase-8 antibody (1 : 50, ab25901; Abcam), or anti-caspase-9 antibody (1 : 100, ab52298; Abcam), followed by incubation with secondary antibodies for 50 min at room temperature. Subsequently, the nuclei were restained using DAPI and analyzed under a microscope (OLYMPUS BX51). Hematoxylin stained the nuclei blue, and DAB produced a brown stain for positive expression of the targeted proteins. DAPI-stained nuclei were blue under UV excitation, and the positive expression of targeted proteins was detected as a red or green light signal, depending on the fluorescein label.

### 2.15. Statistical Analysis

All data were expressed as mean ± SD (standard deviation). The survival rates were analyzed by applying the Kaplan-Meier method, and mean comparisons were performed using the unpaired *t*-test. Statistical analysis was performed using GraphPad Prism 6.0, and the ImageJ 1.42q software was used for quantitative analysis of immunofluorescence images. Statistical significance was set at *p* < 0.05.

## 3. Results

### 3.1. HL-003 Is a Strong Free Radical Scavenger *In Vitro*

**Figure 1:**
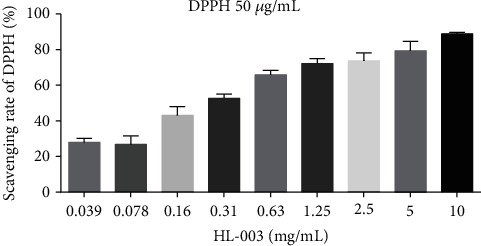
The strong free radical scavenging capability of HL-003 was concentration dependent. Quantitative histogram analysis of free radical scavenging by HL-003 in a cell-free system.

Because HL-003 was designed as an amino mercaptan antioxidant, we initially evaluated its antioxidant capacity *in vitro* using the DPPH assay. The results showed that HL-003 efficiently scavenged DPPH within 30 min in a concentration-dependent manner (Figure 1), which proved that HL-003 is a powerful free radical scavenger with strong antioxidant activity.

### 3.2. HL-003 Has a Better Safety Profile than Amifostine

Since HL-003 was designed and developed as an oral ARS-protective agent, we evaluated its oral safety by an acute toxicity test and a 28-day long-term toxicity test (Tables 1 and 2). The acute toxicity test showed that the maximum tolerated doses (MTDs) of HL-003 and amifostine were 1800 and 600 mg/kg in mice, indicating that HL-003 was better tolerated than amifostine. In the long-term toxicity test, HL-003 had a 28-day MTD of 1000 mg/kg, which indicated that the compound had a strong safety profile. The mortality rate in the acute and long-term toxicity test control groups was 0% (data not shown).

### 3.3. Oral Administration of Different Doses of HL-003 Significantly Improved Survival in Irradiated Mice

**Figure 2:**
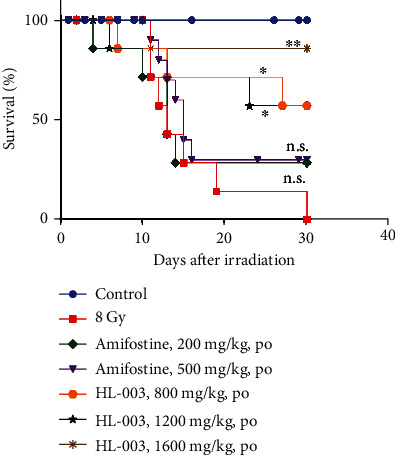
Oral administration of different HL-003 doses improved the survival rate in irradiated mice, whereas oral amifostine had no beneficial effect. Male C57BL/6 mice received oral HL-003 or amifostine 1 or 0.5 h before WAI. Kaplan-Meier survival analysis of mice exposed to 8.0 Gy (^∗^*p* < 0.05, ^∗∗^*p* < 0.01; group size, *n* = 7 or *n* = 10).

To evaluate the potential of HL-003 as an oral ARS-protective agent, we assessed the radioprotective efficacy of different doses of oral HL-003 in male C57BL/6 mice (Figure 2 and Table S2). Thirty days after receiving 8 Gy WBI, the mice in the IR group had a survival rate of 0%. The survival rates in mice exposed to oral amifostine for 30 days were 28.6% and 30% in the amifostine 200 mg/kg and 500 mg/kg groups, respectively. The survival in the amifostine groups did not significantly differ from that in the IR group. Interestingly, oral administration of different HL-003 doses significantly increased the survival of irradiated mice, which had survival rates of 57% (*p* < 0.05), 57% (*p* < 0.05), and 85.71% (*p* < 0.01) in the HL-003 800, 1200, and 1600 mg/kg groups, respectively.

### 3.4. Optimal Time for Oral HL-003 Administration Was 4 h before Irradiation

**Figure 3:**
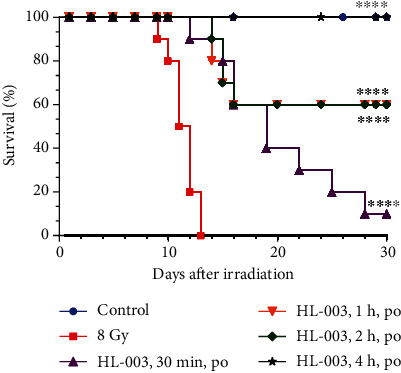
The optimal administration time of oral HL-003 was 4 h before irradiation. The 30-day survival rate of each group was evaluated after oral administration of 1600 mg/kg HL-003 at 0.5, 1, 2, and 4 h before irradiation. Kaplan-Meier survival analysis of mice exposed to 8.0 Gy (^∗∗∗∗^*p* < 0.0001; group size, *n* = 10).

We used the best dose of 1600 mg/kg to determine the optimal time for HL-003 administration in male C57BL/6 mice. In the IR group, the mice began to die 9 days after receiving 8 Gy WBI. The last mouse in this group died 13 days postirradiation, and the 30-day survival rate was 0%. Administration of HL-003 at 0.5, 1, and 2 h before irradiation delayed the death time of the first animal from 9 days postirradiation to 12, 14, and 14 days, respectively, and the 30-day survival rate was 10%, 60%, and 60%, respectively. The survival curves of the HL-003 treatment groups differed significantly from that of the IR group (*p* < 0.0001). More importantly, no mouse died when HL-003 was administered 4 h prior to irradiation, and the survival increased to 100% in irradiated mice. Thus, the optimal HL-003 administration time was 4 h before 8 Gy WBI (Figure 3, Table S3).

### 3.5. Oral Administration of HL-003 Increased the Radiation Tolerance Dose in Mice

**Figure 4:**
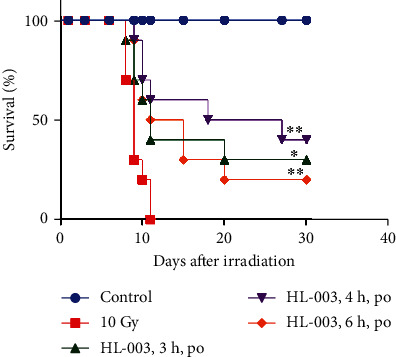
HL-003 increased the survival rate in high-dose irradiated mice. The 30-day survival rate of each group was evaluated after oral administration of 1600 mg/kg HL-003 at 3, 4, and 6 h before irradiation. Kaplan-Meier survival analysis of mice exposed to 10.0 Gy (^∗^*p* < 0.05, ^∗∗^*p* < 0.01; group size, *n* = 10).

In this experiment, we tested whether HL-003 could improve the survival rate in mice under high-dose irradiation. The results showed that administering a dose of 1600 mg/kg HL-003 at 3, 4, and 6 h prior to irradiation significantly improved the survival rate in mice exposed to 10 Gy WBI (*p* < 0.05, Figure 4) Although the survival rate of mice irradiated from 8 Gy to 10 Gy decreased from 100% to 40% under the same administration conditions of HL-003 (1600 mg/kg, 4 h before irradiation), it was improved to some extent under the irradiation dose of 10 Gy. The specific values are shown in Supporting Information Table S4.

### 3.6. Evaluation of PK Parameters of HL-003

We assessed the oral efficacy of HL-003 and evaluated its PK parameters and oral bioavailability in male SD rats (Table 3). The plasma half-life (*T*_1/2_) values for HL-003 after i.v. injection and p.o. administration were 0.67 ± 0.28 and 6.65 ± 2.49 h, respectively. After p.o. administration of 400 mg/kg HL-003, the maximum drug concentration (*C*_max_) in plasma was 21580.4 ± 8971.2 ng/mL, and the time to reach the peak value (*T*_max_) was 0.28 ± 0.21 h. Moreover, the bioavailability of HL-003 was up to 42.29 ± 6.68%.

**Figure 5:**
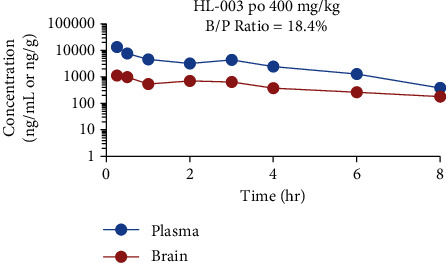
HL-003 concentration in plasma (ng/mL) and brain tissue (ng/g) of ICR mice.

In addition, the BBB was evaluated by ICR mice. The parameters obtained in the experiment were calculated using the Winnonlin 7.0 noncompartment model (Figure 5 and Table S5). The AUC in plasma was 24627 ± 2418 hr^∗^ng/mL, and the AUC in brain tissue was 4536 ± 780 hr^∗^ng/g. The calculated B/P ratio was 18.40 ± 2.23%, indicating that HL-003 could cross into the brain tissue through the BBB.

### 3.7. HL-003 Alleviated Radiation-Induced Morphological Injury of Small Intestine

**Figure 6:**
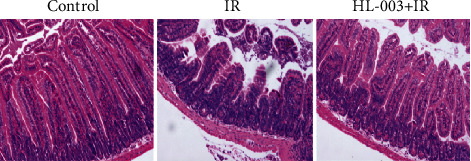
HL-003 improves the morphological changes of small intestine tissue in irradiated mice. Microphotographs show the cross-sectional structure of the small intestine (HE staining).

We evaluated the protective effect of HL-003 on radiation-induced intestinal injury in male C57BL/6 mice to explore the mechanism of radioprotection. We evaluated the effect of HL-003 on the intestinal morphology in mice 3 days after ABI with 15 Gy by HE staining of tissue samples (Figure 6). In contrast to the normal morphology of the nonirradiated intestinal tissue, the small intestinal villi of the irradiated mice were broken, and the crypt was damaged. Oral administration of HL-003 significantly improved the morphological changes in the small intestine of irradiated mice, which resembled that of nonirradiated mice.

### 3.8. HL-003 Promotes Proliferation, Differentiation, and Regeneration of Crypt Cells in Irradiated Male C57BL/6 Mice

**Figure 7:**
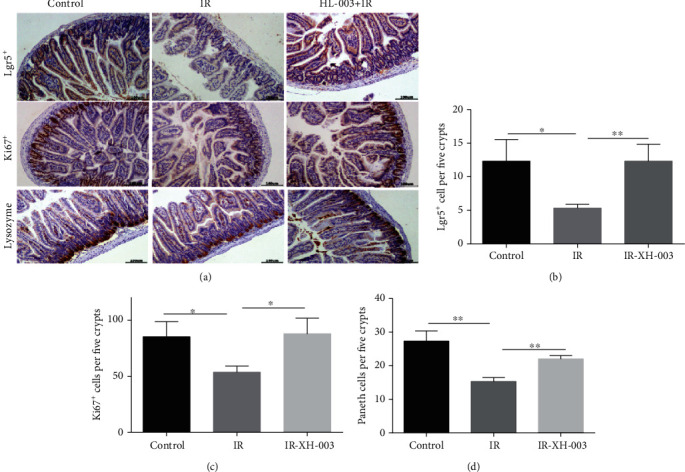
HL-003 promoted the proliferation, differentiation, and regeneration of crypt cells after ABI. (a) Microphotographs of Lgr5^+^, Ki67^+^, and lysozyme^+^ immunohistochemical staining. (b) Quantitative histogram analysis of Lgr5^+^ cells. (c) Quantitative histogram analysis of Ki67^+^ cells. (d) Quantitative histogram analysis of lysozyme^+^ cells. ^∗^*p* < 0.05, ^∗∗^*p* < 0.01, ^∗∗∗^*p* < 0.001, and ^∗∗∗∗^*p* < 0.0001.

The intestinal epithelium can continuously renew itself to maintain homeostasis and rapidly regenerate after injury [23, 24]. In this study, immunohistochemical staining was performed to assess the effect of HL-003 on proliferation and differentiation of crypt cells and its effect on maintaining intestinal cell regeneration. Lgr5^+^ intestinal stem cells (ISCs), lysozyme^+^ Paneth cells, and Ki67^+^ transient amplifying cells (TACs) were evaluated at 3 days after 15 Gy ABI (Figure 7). Compared with the status in the control group, irradiation severely reduced the numbers of Lgr5^+^ ISCs, Ki67^+^ TACs, and lysozyme^+^ Paneth cells in the IR group, whereas these cell types were significantly increased in the small intestinal tissue of irradiated mice treated with HL-003, indicating that HL-003 increased the proliferation, differentiation, and regeneration capability of intestinal crypt cells in these mice.

### 3.9. HL-003 Reduced Apoptosis in Small Intestine Tissue of Irradiated Male C57BL/6 Mice

**Figure 8:**
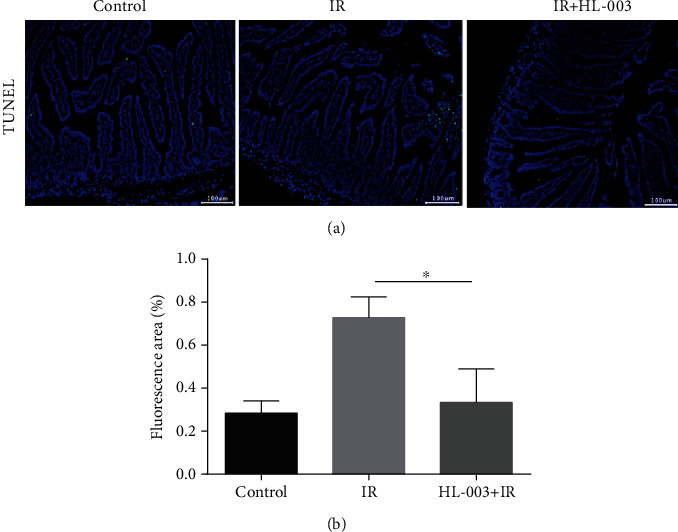
HL-008 reduced the apoptosis of small intestinal cells in irradiated mice. (a) Representative immunofluorescence images assessing apoptosis in tissue samples of the small intestine (green, apoptotic cells). (b) Quantitative histogram analysis of apoptosis (^∗^*p* < 0.05, ^∗∗^*p* < 0.01, ^∗∗∗^*p* < 0.001, and ^∗∗∗∗^*p* < 0.0001).

The effect of HL-003 on apoptosis of small intestine tissue cells in irradiated mice was evaluated by TUNEL analysis (Figure 8). We found that the IR group had more apoptotic small intestinal cells than the control group, but administration of HL-003 significantly reduced the apoptosis of small intestinal cells in irradiated mice, and the difference was statistically significant (*p* = 0.020).

### 3.10. HL-003 Protects DNA Double Strands against Radiation Damage

**Figure 9:**
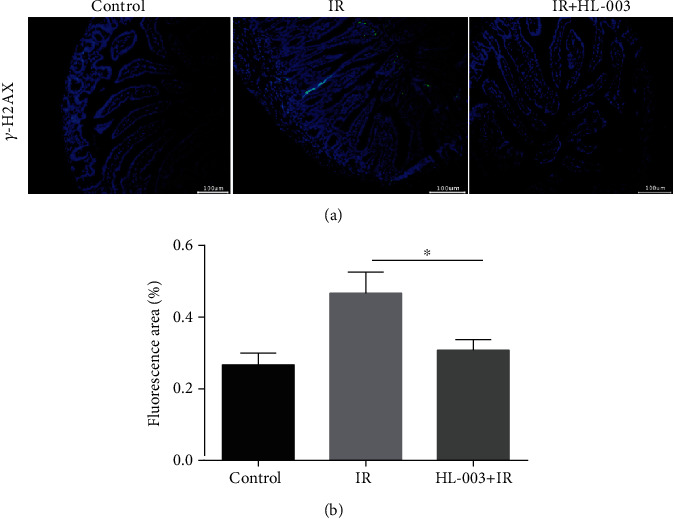
HL-003 suppressed the expression of DNA damage marker protein *γ*-H2AX in small intestinal cells of irradiated mice. (a) Representative immunofluorescence images for the expression of *γ*-H2AX in tissue samples of the small intestine (green, *γ*-H2AX). (b) Quantitative histogram analysis of the *γ*-H2AX positive signal (^∗^*p* < 0.05, ^∗∗^*p* < 0.01, ^∗∗∗^*p* < 0.001, and ^∗∗∗∗^*p* < 0.0001).

To investigate whether HL-003 protected DNA from radiation damage, the expression of the DNA damage marker *γ*-H2AX was analyzed in irradiated male C57BL/6 mice [25]. The results are shown in Figure 9. Compared with the *γ*-H2AX expression status in the control group, the expression of *γ*-H2AX was stimulated in small intestinal cells of irradiated mice, whereas administration of HL-003 suppressed the *γ*-H2AX expression in small intestinal cells of irradiated mice, and the difference was significant (*p* = 0.014). These results suggested that HL-003 alleviated the irradiation-induced DNA damage.

### 3.11. HL-003 Inhibited Intestinal Cell Apoptosis in Irradiated Mice by Suppressing Apoptosis-Related Pathway Proteins

**Figure 10:**
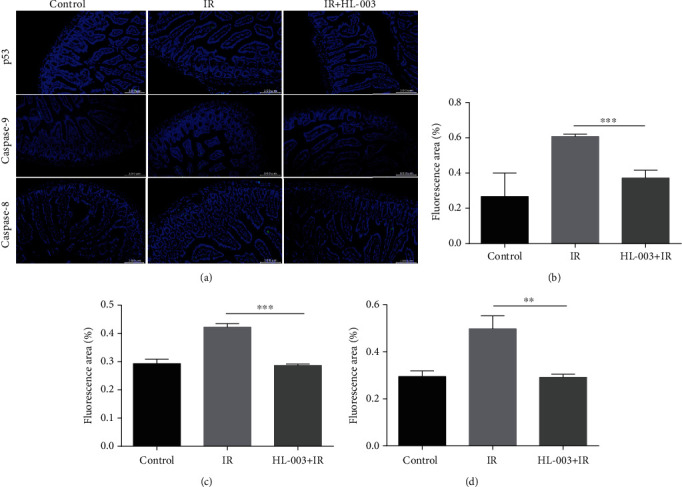
HL-003 suppressed the expression of apoptosis-related proteins p53, caspase-8, and caspase-9 in the small intestine of irradiated mice. (a) Representative immunofluorescence images for the expression of p53, caspase-8, and caspase-9 in tissue samples of the small intestine (green: p53, caspase-9, and -8). (b) Quantitative histogram analysis of p53 positive signal. (c) Quantitative histogram analysis of caspase-9 positive signal. (d) Quantitative histogram analysis of caspase-8 positive signal. (^∗^*p* < 0.05, ^∗∗^*p* < 0.01, ^∗∗∗^*p* < 0.001, ^∗∗∗∗^*p* < 0.0001).

To further study the mechanism of the protective effect of HL-003 on radiation-induced intestinal injury, the expression levels of apoptosis-related pathway proteins p53 and caspase-8/9 were assessed by immunofluorescence (Figure 10). The small intestine of the IR group had higher expression levels of p53 and the caspase-8 and caspase-9 proteins than that of the control group. However, HL-003 treatment of irradiated mice significantly suppressed the expression levels of p53 (*p* = 0.0009), caspase-8 (*p* = 0.003), and caspase-9 (*p* < 0.0001), compared with those levels in irradiated male C57BL/6 mice without further treatment. These results suggested that HL-003 exerted a protective effect on radiation-induced intestinal injury via the p53 pathway and the mitochondrial apoptosis pathway.

## 4. Discussion

To date, the potential radiation hazard has become a serious public health challenge due to the rapid development and wide application of the nuclear industry [26]. Especially radioprotectors should be safe, effective, and easy-to-administer because they should be given to military personnel and first responders prior to entering a known radiation area [27]. During the Cold War, the Walter Reed Army Institute of Research in the United States developed a powerful, highly radioprotective antioxidant containing sulfhydryl groups, named amifostine (or WR2721). However, its application is limited because of its serious side effects, short half-life, nonoral nature, and other disadvantages [28, 29]. The search for a radioprotective agent with a long half-life, oral administration, and no obvious side effects on the human body has always been an important issue in the field of radiation medicine [30, 31].

We previously used the structure of amifostine to design and synthesize a small molecule compound containing a sulfhydryl group, HL-003, for which we have 100% independent intellectual property rights. In the current study, we initially evaluated the antioxidant capacity of HL-003 in a cell-free system *in vitro.* We found that the strong free radical scavenging activity of HL-003 was concentration dependent. The acute toxicity test showed that the safety profile of HL-003 (MTD = 1800 mg/kg) was better than that of amifostine (MTD = 600 mg/kg), and the MDT in the 28-day long-term toxicity test of oral HL-003 for 28 days was 1000 mg/kg, indicating that this compound was a highly safe antioxidant.

By evaluating the effects of different HL-003 doses on the 30-day survival rate in irradiated mice, we observed that oral HL-003 administration improved survival among irradiated mice, whereas oral amifostine did not provide an effective radioprotection. Furthermore, we found that the right time for oral HL-003 administration was 4 h prior to an irradiation dose of 8 Gy. HL-003 still shows a certain radiation protective effect, although the effect under the irradiation dose of 10 Gy is weaker than that of 8 Gy. Therefore, HL-003 is a small molecule radioprotector with a good safety profile, high efficacy, and oral administration.

We further analyzed the PK characteristics of HL-003 *in vivo*. The plasma half-life of HL-003 administered intravenously (*T*_1/2_ = 40.2 ± 16.8 min) and orally (*T*_1/2_ = 6.65 ± 2.49 h) was longer than that of amifostine (*T*_1/2_ = 10 min). Its oral bioavailability was as high as 42.29%. Interestingly, HL-003 can cross the BBB and has a potential radioprotective effect on brain radiation damage. These results show that HL-003 is a radioprotective agent with a favorable safety profile, high efficacy, oral administration, and a long half-life. Specifically, in our preclinical studies, HL-003 had higher radioprotective efficacy than amifostine.

To further analyze the radioprotective properties of HL-003 in our study, its mechanism of protection against radiation-induced intestinal injury was assessed. The small intestine is very susceptible to irradiation, and radiotherapy patients are often affected by severe intestinal reactions, such as nausea, vomiting, and diarrhea, which seriously diminish the quality of life in those patients [32]. Unfortunately, there is no specific drug for radiation-induced intestinal injury in clinical use [33]. Hence, the development of effective radioprotectors is a critical goal. Staining of intestinal tissue samples with HE revealed that HL-003 significantly improved the intestinal morphological changes in irradiated mice, indicating that it was effective against radiation-induced intestinal injury.

The intestinal epithelium is the fastest self-renewing tissue in mammals that depends on self-renewal by intestinal epithelial stem cells located in the crypts to maintain the dynamic balance with the environment and to regenerate quickly after damage [34, 35]. ISCs renew themselves by expressing Lgr5, which is an intestinal stem cell marker. The Lg5^+^ ISCs are a known key factor in intestinal regeneration after radiation injury [36, 37]. In addition, Paneth cells, which are located at the bottom of crypt cells, maintain homeostasis of the intestinal environment by secreting lysosomes and other antibacterial proteins [38]. Interestingly, there is a positive correlation between the number of Paneth cells and the number of Lgr5^+^ stem cells [39]. The hyperplastic cells of the small intestinal epithelium can be identified due to the expression of the proliferation marker protein Ki67 [40]. We found that HL-003 significantly prevented the irradiation-induced decrease in Lgr5^+^ ISCs, Ki67^+^ TACs, and lysozyme^+^ Paneth cells, and it promoted the regeneration, differentiation, and proliferation of intestinal epithelial cells and maintained homeostasis of the intestinal epithelium.

In this study, TUNEL staining analysis showed that HL-003 significantly reduced the apoptosis in the small intestine of irradiated mice. Immunofluorescence analysis was performed to explore the pathway related to apoptosis. It is known that radiation can directly induce DNA damage through ROS production, which mediates the activation of apoptosis-related pathways that can result in the loss of tissue and organ function [41]. Phosphorylated histone H2AX is an important marker of DNA double-strand breaks [42], which can promote the phosphorylation of p53, a proapoptotic factor [43]. Specifically, activated p53 can regulate DNA damage repair and cell cycle checkpoints. Moreover, it can promote the release of cytochrome c from the mitochondria, activate the caspase cascade, and induce cell apoptosis [44]. HL-003 treatment significantly lowered the levels of phosphorylated histone H2AX, phosphorylated p53, and activated caspase-8 and caspase-9 in the intestine of mice after irradiation, indicating that HL-003 significantly reduced radiation-induced DNA damage, inhibited the activation of p53, blocked the caspase cascade reaction, and protected the small intestine from radiation injury.

## 5. Conclusion

In this study, we built on our earlier work on the small molecule antioxidant agent HL-003 by comprehensively evaluating the compound's safety, pharmacokinetics, and efficacy. We proved that HL-003 is an ideal radioprotective agent with a promising safety profile, high efficacy, oral administration, and a long half-life. We further investigated the mechanism of radioprotection by HL-003 against radiation-induced intestinal injury. We found that HL-003 provided radioprotection by promoting the proliferation, differentiation, and regeneration of the intestinal epithelium, protecting DNA from radiation damage, and inhibiting the activation of apoptosis-related pathways. Thus, HL-003 is a potential small molecule radioprotector.

## Figures and Tables

**Table 1:** Acute toxicity test of HL-003 and amifostine in C57BL/6 mice.

mg/kg	Survival rate								
	600	700	800	1200	1600	1800	2000	2400	3200
Amifostine	100% (6/6)	83.33% (5/6)	66.67% (4/6)	NA	NA	NA	NA	NA	NA
HL-003	NA	NA	NA	100% (10/10)	100% (10/10)	100% (10/10)	50% (5/10)	40% (4/10)	0% (0/10)

NA: not available. NA indicates that no acute toxicity test was conducted at this dose. (*n*_1_/*n*_2_): *n*_1_ represents the number of surviving mice 14 days postadministration and *n*_2_ represents the number of mice in each group preadministration.

**Table 2:** 28-day long-term toxicity test of HL-003 in C57BL/6 mice.

mg/kg	Survival rate			
	800	1000	1200	1600
HL-003	100% (10/10)	100% (10/10)	80% (8/10)	50% (5/10)

(*n*_1_/*n*_2_): *n*_1_ represents the number of surviving mice 14 days postadministration and *n*_2_ represents the number of mice in each group preadministration.

**Table 3:** PK parameters of HL-003 in SD rats. SD rats (group size, *n* = 3) received an HL-003 dose of 100 mg/kg i.v. or 400 mg/kg p.o., and blood samples were collected from 0 to 24 h for PK parameter analysis.

PK parameter	Mean	Standard deviation
PK parameters of HL-003 in male SD rats after a dose of 100 mg/kg i.v.		
HL_Lambda_z (*T*_1/2_, h)	0.67	0.28
*C*_max_ (ng/mL)	77510.6	2202.1
AUC_last_ (h^∗^ng/mL)	30589.1	1199.2
AUCINF_pred (h^∗^ng/mL)	30606.8	1198.3
MRTlast (h)	0.32	0.05
Vz_pred (L/kg)	3.15	1.26
Cl_pred (L/h/kg)	3.27	0.13
*Λ*z calculation time range (h)	NA	NA

PK parameters of HL-003 in male SD rats after a dose of 400 mg/kg p.o.		
HL_Lambda_z (*T*_1/2_, h)	6.65	2.49
*T*_max_ (h)	0.28	0.21
*C*_max_ (ng/mL)	21580.4	8971.2
AUC_last_ (h^∗^ng/mL)	51744.1	8389.0
AUCINF_pred (h^∗^ng/mL)	55842.4	6086.6
MRTlast (h)	5.71	0.70
Vz_F_pred (L/kg)	70.55	32.64
Cl_F_pred (L/hr/kg)	7.22	0.82
*λ*z calculation time range (h)	NA	NA
*F* (%)	42.29	6.86

NA: not available. NA means that the data has not been calculated.

## Data Availability

The data used to support the findings of this study are available from the corresponding author upon request.

## Supplementary Materials

Supplementary materials There are five tables included in the supplementary materials: The data for average percentage of DPPH scavenged by HL-003 at different concentrations is shown in Table S1; the data for radioprotective efficiency of HL-003 and amifostine orally administrated after 8 Gy irradiation is shown in Table S2; the data for radioprotective effect of HL-003 at different administration time points after 8 Gy irradiation is shown in Table S3; the data for radioprotective effect of HL-003 at different administration time points after 10 Gy irradiation is shown in Table S4; and the PK parameters of HL-003 in ICR mice were shown in Table S5.
