# Supplementary material for: Preclinical Evaluation of Safety, Pharmacokinetics, Efficacy, and Mechanism of Radioprotective Agent HL-003
Source: Oxid Med Cell Longev. 2021 Feb 19;2021:6683836. doi: 10.1155/2021/6683836 (PMC7914087; doi:10.1155/2021/6683836)
Supplement: Supplementary materials — There are five tables included in the supplementary materials: The data for average percentage of DPPH scavenged by HL-003 at different concentrations is shown in Table S1; the data for radioprotective efficiency of HL-003 and amifostine orally administrated after 8 Gy irradiation is shown in Table S2; the data for radioprotective effect of HL-003 at different administration time points after 8 Gy irradiation is shown in Table S3; the data for radioprotective effect of HL-003 at different administration time points after 10 Gy irradiation is shown in Table S4; and the PK parameters of HL-003 in ICR mice were shown in Table S5. [file 6683836.f1.docx]

**Supplementary materials**

# Preclinical Evaluation of Safety, Pharmacokinetics, Efficacy, and Mechanism of Radioprotective Agent HL-003

Yahong Liu, Longfei Miao, Yuying Guo, Hongqi Tian

Tianjin Key Laboratory of Radiation Medicine and Molecular Nuclear Medicine, Institute of Radiation Medicine, Peking Union Medical College and Chinese Academy of Medical Science, Tianjin 300192, China.

Correspondence should be addressed to Hongqi Tian: [tianhongqi@irm-cams.ac.cn](mailto:tianhongqi@irm-cams.ac.cn).

# Yahong Liu and Longfei Miao are the co-first authors of this research paper

Table S1 The average percentage of DPPH scavenged by HL-003 at different concentrations.

| HL-003 (mg/mL) | Mean (%) | Standard Deviation |
| --- | --- | --- |
| 0 | 0 | 0 |
| 0.039 | 27.85224247 | 2.38175534 |
| 0.078 | 26.81113869 | 4.82376527 |
| 0.16 | 43.15190655 | 4.872923052 |
| 0.31 | 52.64006043 | 2.397072428 |
| 0.63 | 65.82501441 | 2.561839427 |
| 1.25 | 72.09060118 | 2.832031167 |
| 2.5 | 73.6639207 | 4.492177764 |
| 5 | 79.31590312 | 5.347237633 |
| 10 | 88.7812104 | 0.83069551 |

Table S2 Data for radioprotective efficiency of HL-003 and amifostine orally administrated after 8 Gy irradiation.

| Group | Dose mg/kg | Survival number | Death rate | p value |
| --- | --- | --- | --- | --- |
| Control | - | 10/10 | 0% | - |
| IR | - | 0/7 | 100% | - |
| Amifostine | 200 | 2/7 | 71.42% | 0.559 |
| Amifostine | 500 | 3/10 | 60% | 0.318 |
| HL-003 | 800 | 4/7 | 28.57% | 0.033 |
| HL-003 | 1200 | 4/7 | 28.57% | 0.047 |
| HL-003 | 1600 | 6/7 | 14.29% | 0.002 |

Treatment group vs IR group, *p<0.05, **p<0.01, ***p<0.001

Table S3 Data for radioprotective effect of HL-003 at different administration time points after 8 Gy irradiation.

| Group | Time | Survival number | Death rate | p value |
| --- | --- | --- | --- | --- |
| Control | - | 10/10 | 0% | - |
| IR | - | 0/10 | 100% | - |
| HL-003 | 0.5 h | 1/10 | 90% | <0.0001 |
| HL-003 | 1 h | 6/10 | 40% | <0.0001 |
| HL-003 | 2 h | 6/10 | 40% | <0.0001 |
| HL-003 | 4 h | 10/10 | 0% | <0.0001 |

Treatment group vs IR group, *p<0.05, **p<0.01, ***p<0.001

Table S4 Data for radioprotective effect of HL-003 at different administration time points after 10 Gy irradiation.

| Group | Time | Survival number | Death rate | p value |
| --- | --- | --- | --- | --- |
| Control | - | 10/10 | 0% | - |
| IR | - | 0/10 | 100% | - |
| HL-003 | 3 h | 3/10 | 70% | 0.02 |
| HL-003 | 4 h | 4/10 | 60% | 0.001 |
| HL-003 | 6 h | 2/10 | 80% | 0.003 |

Treatment group vs IR group, *p<0.05, **p<0.01, ***p<0.001

Table S5 PK parameters of HL-003 in ICR mice. ICR mice (group size, n=3) received an HL-003 dose of 400 mg/kg po, and blood samples and brain tissue were collected at 15min、30min、1h、2h、3h、4h、6h、8h for BBB analysis.

| PK parameter of HL-003 in plasma | | |
| --- | --- | --- |
| PK parameter | Mean | Standard Deviation |
| T_1/2_, h | 1.48 | 0.33 |
| T_max_ (h) | 0.25 | 0.14 |
| C_max_ (ng/ml) | 13470 | 10125 |
| AUC_0-t_ (hr*ng/ml) | 23816 | 2420 |
| AUC0-∞ (hr*ng/ml) | 24627 | 2418 |
| PK parameter of HL-003 in brain | | |
| PK parameter | Mean | Standard Deviation |
| T_1/2_, h | 3.36 | 0.42 |
| T_max_ (h) | 0.25 | 0.14 |
| C_max_ (ng/ml) | 1126 | 795 |
| AUC_0-t_ (hr*ng/ml) | 3663 | 695 |
| AUC0-∞ (hr*ng/ml) | 4536 | 780 |
| B/P(%) | 18.40% | 2.23% |

B/P Ratio=AUC_(brain)_/AUC_(plasma)_
